# Familial Immune Thrombocytopenia Associated With a Novel Variant in *IKZF1*

**DOI:** 10.3389/fped.2019.00139

**Published:** 2019-04-24

**Authors:** Panida Sriaroon, Yenhui Chang, Boglarka Ujhazi, Krisztian Csomos, Hemant R. Joshi, Qin Zhou, Devin W. Close, Jolan E. Walter, Attila Kumánovics

**Affiliations:** ^1^Division of Allergy, Immunology, and Rheumatology, Department of Pediatrics, University of South Florida Morsani College of Medicine, St. Petersburg, FL, United States; ^2^Pathology and Laboratory Medicine, Johns Hopkins All Children's Hospital, St. Petersburg, FL, United States; ^3^Department of Pathology, University of Utah School of Medicine, Salt Lake City, UT, United States; ^4^ARUP Laboratories, Institute for Clinical and Experimental Pathology, Salt Lake City, UT, United States; ^5^Division of Allergy/Immunology, Massachusetts General Hospital for Children, Boston, MA, United States; ^6^Department of Laboratory Medicine and Pathology, Mayo Clinic, Rochester, MN, United States

**Keywords:** primary immunodeficiency, autoimmunity, immune thrombocytopenia, ITP, IKAROS deficiency, *IKZF1*

## Abstract

We report a novel variant in *IKZF1* associated with IKAROS haploinsufficiency in a patient with familial immune thrombocytopenia (ITP). IKAROS, encoded by the *IKZF1* gene, is a hematopoietic zinc-finger transcription factor that can directly bind to DNA. We show that the identified *IKZF1* variant (p.His195Arg) alters a completely conserved histidine residue required for the folding of the third zinc-finger of IKAROS protein, leading to a loss of characteristic immunofluorescence nuclear staining pattern. In our case, genetic testing was essential for the diagnosis of IKAROS haploinsufficiency, of which known presentations include infections, aberrant hematopoiesis, leukemia, and age-related decrease in humoral immunity. Our family study underscores that, after infections, ITP is the second most common clinical manifestation of IKAROS haploinsufficiency.

## Case Report

We identified a 14-year-old Caucasian male, who at age 4 years presented with treatment-refractory immune thrombocytopenia (ITP) requiring several months of treatment with corticosteroids and high dose intravenous immunoglobulin (IVIG). He also had a history of recurrent otitis requiring tympanostomy tube placement and adenoidectomy. Laboratory studies identified low serum immunoglobulin (Ig) levels and vaccine titers with normal B and T cell numbers. Anti-platelet antibodies were not detected. Bone marrow examination showed increased number of megakaryocytes without other abnormalities. At that time, he was given a diagnosis of common variable immunodeficiency (CVID). Over the next decade, thrombocytopenia (ranging 28,000–114,000/mm^3^) and dysgammaglobulinemia persisted but he was clinically asymptomatic and without major infections. Laboratory data at age 14 years showed normal total B and T cell numbers but low NK cells, class-switched B cells, and CD4/CD8 ratio (Table 1).

**Table 1:** Clinical and laboratory data.

		**Patient**		**Mother (age 44)**	**Maternal grandfather (age 75)**
		**Age 4**	**Age 14**		
Age of onset		4 years		21 years	–
Infections		–	–	–	–
Clinical findings		Thrombocytopenia	Thrombocytopenia	Thrombocytopenia (21–24 years): small ischemic stroke in cerebellum and thrombosis of a vertebral artery (42 years)	Healthy
Treatment		Systemic steroids, high dose IgG	None	Splenectomy (24 years)	None
**CELL COUNT (/mm^3^)**					
Neutrophil (/mm^3^)		2,400	1,800 (1,500–6,600)	3,478 (1,500–7,800)	1,500 (1,400–7,000)
Lymphocyte (/mm^3^)		2,750	2,000 (1,000–3,500)	2,033 (850–3,900)	1,700 (700–3,100)
Platelets (/uL)		**↓ 2,300** (189K−403K)	**↓ 114,000** (150K−450K)	337,000 (140K−400K)	204,000 (150K−379K)
Anti-platelet antibody		Negative	Negative	Negative	ND
**Ig (mg/dL)**					
IgG		**↓ 444** (528–2,190)	**↓ 506** (768–1632)	997 (768–1632)	907 (768–1632)
IgA		**↓ 41** (61–345)	**↓ 29** (68–408)	**↓ 54** (68–408)	**↓ 57** (68–408)
IgM		59 (48–226)	**↓ 10** (35–263)	**↓ 21** (35–263)	63 (35–263)
**LYMPHOCYTE COUNT (/mm**^**3**^**)**					
B cells	Total CD19+	165	243 (120–740)	**↓ 87** (91–610)	**↓ 46** (74–510)
	Total memory (CD19+CD27+)	ND	**↓ 47** (50–200)	14 (13–148)	**↓ 6** (13–148)
	Class Switched memory (CD19+CD27+IgD-IgM-)	ND	**↓ 2** (30–110)	**↓ 1** (4–62)	**↓ 3** (4–62)
T cells	Total CD3+	1,470	1,408 (850–3200)	1,305 (570–2400)	1,463 (660–2,200)
	CD8+	809	557 (300–1300)	683 (210–1200)	992 (150–1,050)
	CD4+	514	707 (400–2100)	601 (430–1800)	462 (490–1,600)
	CD4+CD45RA+	ND	298 (230–1400)	**↓ 166** (350–1100)	**↓ 28** (260–1,000)
	CD4+CD45R0+	ND	411 (160–700)	469 (340–1150)	**↓ 433** (490–1,200)
	CD4/CD8 ratio	**↓ 0.64** (1.17–2.94)	1.27 (1.06–2.26)	**↓ 0.88** (1–3.20)	**↓ 0.44** (0.86–5)
	Proliferation	Normal (PHA, Con A, PWM)	Normal (PHA, Con A, PWM)	Normal (PHA, Con A, PWM, anti-CD3-CD28)	ND
NK cells	CD16+CD56+	127 (102–945)	**↓ 55** (92–1200)	190 (78–470)	209 (78–470)

*Numbers in parenthesis indicate age-adjusted reference range. Values in bold are outside of reference range. Arrows pointing up and down indicate increased and decreased values, respectively. Ig, immunoglobulin; ND, not determined; PHA, phytohemagglutinin; Con A, Concanavalin A; PWM, Pokeweed mitogen; NK, natural killer*.

The patient's mother had severe ITP diagnosed at age 21 and required several treatments including splenectomy at age 24. Her ITP resolved and never recurred. At age 42, she developed a small ischemic stroke in the cerebellum and was found to have thrombosis of a vertebral artery. There was no evidence of coagulopathy. She had low levels of IgA, IgM, total B cells, switched memory B cells, and naïve CD4 T cells (Table 1). Despite the measurable immune dysfunction, she never had severe or recurrent infections.

**Figure 1:**
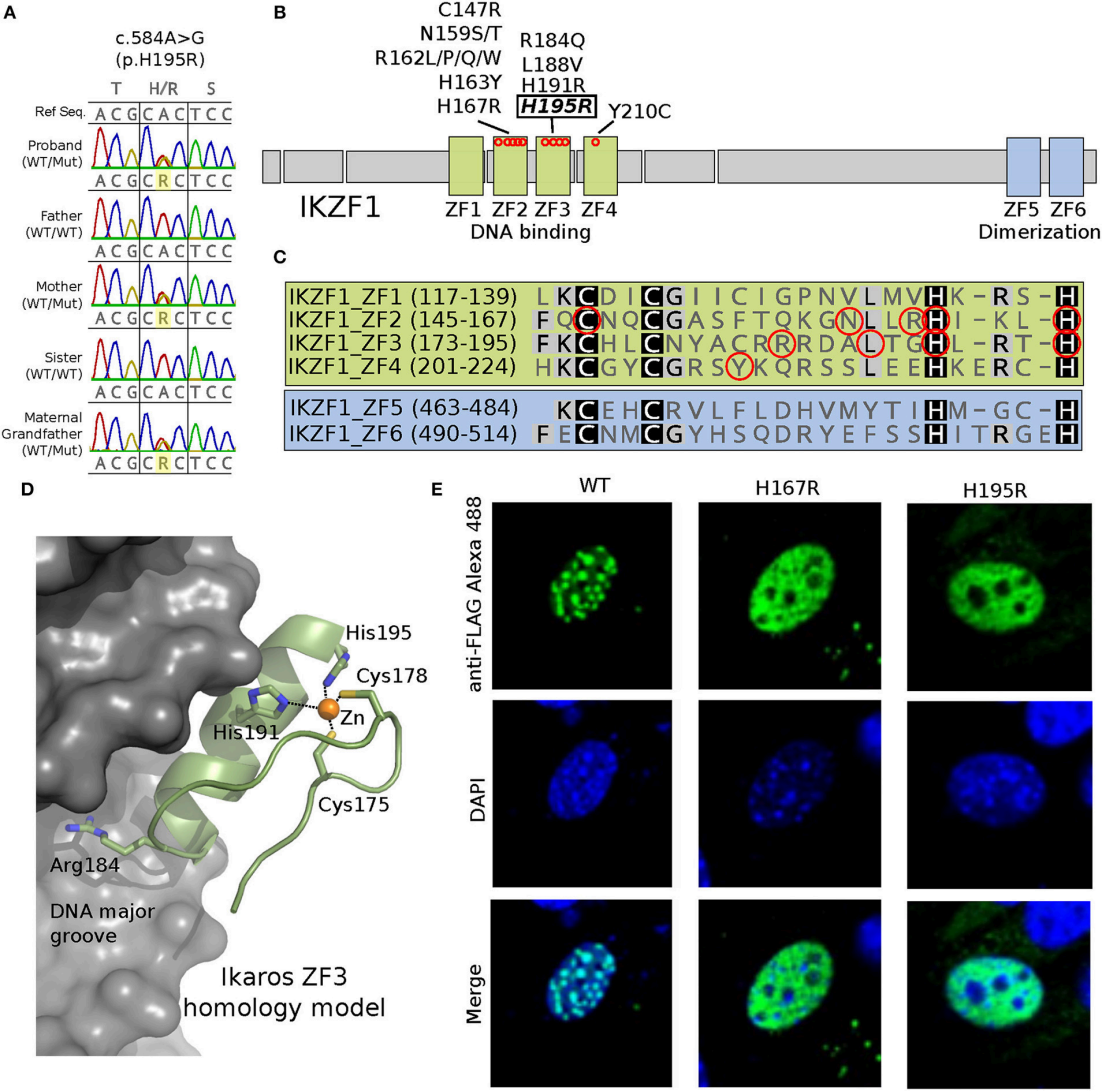
A novel c.584A>G/p.His195Arg variant is observed in affected family members and results in a protein where a highly conserved position critical for C2H2-type Zinc Finger domain folding is altered (H195R). **(A)** Sanger sequencing data showing the heterozygous c.584A>G genotype (WT/Mut) observed in the proband, his mother and maternal grandfather; his father and sister are unaffected (WT/WT). **(B)** Schematic representation of the IKAROS protein showing the 4 DNA binding and 2 dimerization zinc finger (ZF) domains. Approximate locations along the primary sequence for known missense variants are shown as red circles. **(C)** Primary protein sequence alignment of the 6 ZF domains. Conserved Cys and His residues that distinguish this class of C2H2-type ZF domains are highlighted. Known disease associated variant positions are indicated with red circles. **(D)** A homology model for the IKAROS ZF3 bound to DNA was constructed using SWISS-MODEL (1) (swissmodel.expasy.org) using PDB ID 1P47 as template. The model highlights positions of disease associated variants in ZF3, which typically contact the DNA substrate (Arg184) or coordinate zinc (His191 and His195). **(E)** Expression of the FLAG epitope-tagged H195R IKAROS variant in NIH 3T3 fibroblasts results in a loss of the punctate pericentromeric heterochromatin staining within the nucleus (images were acquired at 600x magnification); a phenotype observed for previously reported IKAROS variants such as H167R (2, 3). Wild type and mutant proteins were made the following way. A pcDNA3.1+/C-(K)DYK vector containing a wild-type version of *IKZF1* transcript NM_006060 (Clone ID OHu28071) was purchased from GenScript. Using this as template, vectors containing the H167R or H195R point mutations were generated using a Q5 Site-directed mutagenesis kit (New England Biolabs) following the manufacturer's protocol. The following primer pairs were used for mutagenesis: IKZF1_H167R_F 5′-ATCAAGCTGCGTTCCGGGGAG-3′ and IKZF1_H167R_R 5′- GTGCCGGAGCAGGTTGCC; IKZF1_H195R_F 5′- CTGAGGACGCGCTCCGTTGGTAAAC and IKZF1_H195R_R 5′- GTGGCCAGTGAGGGCGTC-3′. NIH3T3 cells were transfected with the IKAROS plasmids using TransIT-X2®-3T3 transfection kit (Mirus Bio). After 24 h post-transfection, the cells were seeded onto poly-L-lysine treated cover slips. The next day, the cells were washed, fixed 4% paraformaldehyde and permeabilized with 0.1% Triton X-100. The cells were then incubated with rabbit anti-FLAG antibody (SIGMA), or normal rabbit IgG (Santa Cruz) and with Alexa Fluore 488 goat anti-rabbit IgG secondary antibody (Life Technologies). Cells were washed and counter stained with DAPI (Invitrogen). Washed cells were mounted on slides using ProLong Diamond Antifade Mountant (ThermoFisher). Images were acquired with a NiKon A1R^+^ confocal microscope with a 60x oil immersion objective (Nikon Instruments Inc., Melville, NY).

An in-house next generation sequencing (NGS) panel of 180 primary immunodeficiency-associated genes identified a variant in *IKZF1* in the patient. In both the index case and his mother, genetic evaluation by Sanger sequencing verified novel heterozygous missense variant in the DNA-binding zinc finger (ZF) 3 domain of *IKZF1* (c.584A>G, p.His195Arg) (Figure 1A). The same mutation was detected in the unaffected maternal grandfather, who, at age 75, was healthy and had no history of recurrent infections or thrombocytopenia. The grandfather had low levels of IgA, total B cells, and naïve CD4 T cells (Table 1). Interestingly, CD4/CD8 ratio and class switched memory B cells were markedly low in all three family members.

## Functional Evaluation of Novel *IKZF1* Mutation

IKAROS is a zinc-finger protein, in which two cysteines and two histidines coordinate zinc required for protein folding and function (Figures 1B–D). IKAROS binds pericentromeric DNA and recruits the Nucleosome Remodeling and histone Deacetylase (NuRD) complex to lymphoid lineage genes to enhance their chromatin accessibility and transcription (4). The p.His195Arg variant identified in the family alters one of the obligate zinc-coordinating histidines of the zinc-finger fold. To experimentally confirm the pathogenicity of p.His195Arg, we performed an *in vitro* functional study of IKAROS protein. Epitope-tagged mutant IKAROS proteins (Figures 1B–D) expressed in NIH3T3 cells showed an immunofluorescent staining characteristic of loss of function (Figure 1E), consistent with previous findings of other pathogenic mutations in *IKZF1* (2, 3).

## Discussion

IKAROS is a hematopoietic zinc-finger transcription factor encoded by the *IKZF1* gene. IKAROS haploinsufficiency can present with infections, autoimmunity, leukemia, or patients can be clinically asymptomatic. Germline mutations in *IKZF1* lead to T cell malignancies in mice, whereas somatic mutations in human mark high-risk B-progenitor acute lymphoblastic leukemia (B-ALL). Patients with heterozygous germline loss-of-function (LOF) *IKZF1* variants mainly present with autosomal dominant antibody deficiencies (B-cell defects and dysgammaglobulinemia) and abnormal hematopoiesis (3, 5). In previous reports, their presentations varied from being asymptomatic to recurrent infections, autoimmunity, cytopenias, and T- or B-ALL (3–9). The increased risk of autoimmunity in IKAROS haploinsufficiency might be explained by the lower threshold for activation caused by decreased CD22 expression on mutant B lymphocytes (9). Germline dominant negative mutations (DN) have been linked to early-onset combined immunodeficiency and B-ALL (7, 10).

We report a novel *IKZF1* variant (p.His195Arg) in a family. This variant is likely to be loss-of-function, similar to previously described pathogenic variants associated with recurrent infections and autoimmunity (3) (Figure 1E). Both the proband and his mother first presented with profound thrombocytopenia at ages 4 and 21 years, respectively, and were without major infections despite having dysgammaglobulinemia and skewed B-cell compartments. In this family, the degree of B-cell loss increases with age, which is consistent with previous reports (3). Interestingly, the 75-year-old grandfather was asymptomatic despite having B-cell lymphopenia. The reason for the discrepancy between the degree of humoral immune defect and clinical presentation in patients with *IKZF1* mutations remains unknown. Although splenectomy may have contributed to the immune deficiency seen in the proband's mother (11, 12), we feel this unlikely to be substantial, as the overall immunological phenotype seen in all three mutation carriers is highly similar among each other and to previously described IKAROS deficiency cases (3).

Together with our patients, to date approximately 60 cases from 19 unrelated families have been reported to have 14 unique germline heterozygous *IKZF1* mutations, including missense, splice site, frameshift, partial- and whole-gene deletions (3, 5–9, 13) (Figure 1B). The most common primary clinical presentation is recurrent infections (25/60) and is followed by hematologic and autoimmune manifestations. Four familial cases were reported to have B-ALL, and one with T-ALL (3, 4, 7). Eighteen of 60 mutation carriers (30%) identified by family studies were asymptomatic at the time of reporting, suggesting incomplete penetrance (3, 13).

Familial ITP is uncommon (14) and other causes of thrombocytopenia including genetic defects should be considered when more than one family members have a history of ITP. Early diagnosis of the underlying condition is critical, as it can alter therapeutic approach and outcome. Currently, there are no screening tests for IKAROS haploinsufficiency. Due to heterogeneous presentations, most *IKZF1* mutations are serendipitously identified using exome sequencing or gene panels. As described here, patients with *IKZF1* mutation may not present with infections and therefore immune evaluation might be delayed. The diagnosis of IKAROS haploinsufficiency in our family would have been missed if immune evaluation with Ig levels had not been obtained during the thrombocytopenia evaluation in the index case. Therefore, we recommend that quantitative Ig levels be included in the initial workup in all ITP cases (15).

## Concluding Remarks

We identified a novel germline heterozygous pathogenic variant in *IKZF1* in a single family whose affected members' initial presentation was severe chronic thrombocytopenia. Together with previous reports, this study suggests that autoimmunity, particularly ITP, is the second most common clinical presentation (~20%) of heterozygous LOF *IKZF1* defects after infections (~40%). ITP is reported in 7–14% of CVID patients, and in ~0.01% of general population (16). In patients with refractory thrombocytopenia, especially those with family history of cytopenias or autoimmune diseases, we suggest immunologic evaluation even in the absence of infections. Although the reason for the highly variable clinical presentation of IKAROS deficiency remains unknown, determining the genetic diagnosis can help anticipate complications in patients with IKAROS deficiency, such as the increased risk of developing aberrant hematopoiesis, leukemia, and age-related decrease in humoral immunity.

## Ethics Statement

This study was carried out in accordance with the recommendations of Johns Hopkins School of Medicine Institutional Review Board with written informed consent from all subjects. All subjects gave written informed consent in accordance with the Declaration of Helsinki. The protocol was approved by the Johns Hopkins School of Medicine Institutional Review Board.

## Author Contributions

YC, BU, KC, HJ, QZ, and DC performed experiments. PS and DC made the figures and tables. PS, JW, and AK designed the experiments and wrote the paper with input from all other authors.

### Conflict of Interest Statement

The authors declare that the research was conducted in the absence of any commercial or financial relationships that could be construed as a potential conflict of interest.
